# Apport de l’IRM dans la prise en charge des compressions médullaires lentes non traumatiques

**DOI:** 10.11604/pamj.2016.24.221.8525

**Published:** 2016-07-12

**Authors:** Nfally Badji, Hamidou Deme, Geraud Akpo, Boucar Ndong, Mouhamadou Hamine Toure, Sokhna Ba Diop, El Hadji Niang

**Affiliations:** 1 Service de Radiologie Générale CHU Aristide Le Dantec, Dakar, Sénégal; 2 Service de Radiologie Générale CHUN FANN, Dakar, Sénégal

**Keywords:** IRM, compressions médullaires lentes, épidurites infectieuses, épidurites métastatiques, MRI, slow spinal cord compressions, infectious epiduritis, metastatic epiduritis

## Abstract

Les compressions médullaires lentes sont dues au développement dans le canal médullaire d’une lésion expansive. C’est une pathologie très fréquente dont le diagnostic est essentiellement clinique. L’imagerie par résonnance magnétique occupe une place incontournable dans le diagnostic de localisation et la recherche étiologique. En Europe l’étiologie tumorale est prépondérante. Le but de cette étude était de décrire les aspects IRM des compressions médullaires lentes et de déterminer le profil étiologique. Il s’agit d’une étude rétrospective portant sur 97 observations colligées au service de radiologie du CHUN de Fann sur une période de 30 mois (du 08/03/10 au 29/09/12). On été inclus dans l’étude, tous les patients adressés pour un tableau de compression médullaire lente survenu dans un contexte non traumatique. L’âge moyen des patients était de 42,6 ans avec des extrêmes compris entre 04 mois et 85 ans. Nous avons étudié la topographie des lésions (étage rachidien, compartiments canalaires) leur rehaussement et les critères d’orientation étiologique. Le protocole d’examen permettait la réalisation de séquence pondérées T1 sans avec injection de gado, T2, STIR et T2 DRIVE centrées sur les niveaux lésionnels ou les zones suspectes. L’IRM a permis de préciser le siège exact et l’étendue des lésions. L’atteinte du rachis dorsal représentait 42% des cas, suivi du rachis cervical avec 32% des cas. Les atteintes lombo-sacrées et pluri-étagées représentaient respectivement 18% et 08% des cas. Les lésions extradurales représentaient 87% des cas, suivi des lésions intradurales extramédullaires avec 08% des cas et des lésions intramédullaires dans 05% des cas. La particularité du profil étiologique de notre étude est la prédominance des épidurites infectieuses et la fréquence relative des épidurites métastatiques comparée aux séries occidentales. L’IRM vertébro-médullaire occupe une place capitale dans le diagnostic positif, topographique et étiologique des compressions médullaires.

## Abstract (en)

Slow spinal cord compressions are due to the development of an expansive lesion within the medullary canal. This is a very common condition whose diagnosis is primarily clinical. The magnetic resonance imaging occupies a central and currently irreplaceable position in diagnosis and localization as well as in etiological research. Etiology of cancer is predominant in Europe. This study aims to describe the MRI features of slow spinal cord compressions and to determine its etiologic profile. This is a retrospective study of 97 cases whose data were collected at the Department of Radiology, National University Hospital Centre (CHUN) of Fann, over a period of 30 months (from March 8, 2010 to September 29 2012). All patients referred for slow spinal compression occurred in a non traumatic context were included in the study. The average age of patients was 42.6 years, ranging between 04 months and 85 years. We studied the topography of lesions (spinal floor, ductal compartments), their enhancement and their etiological criteria. The review protocol allowed the realization of T1-weighted sequences with no injection of gado, T2-weighted sequences, STIR sequences and T2-weighted DRIVE sequences centered on lesions levels or suspicious areas. MRI allowed to determine the exact location and the extent of lesions. The involvement of the thoracic spine occurred in 42% of cases, followed by the cervical spine in 32% of cases. The lumbosacral damages and multi-stage damages were found in 18% and 8% of cases respectively. Extradural lesions were found in 87% of cases, followed by intradural extramedullary lesions in 08% of cases and intramedullary lesions in 05% of cases. The peculiarity of etiologic profile of our study is the prevalence of infectious epiduritis and the relative frequency of metastatic epiduritis compared to the Western series. The vertebral-medullary MRI occupies a crucial place in the positive, topographic and etiological diagnosis of spinal cord compressions.

## Introduction

Les compressions médullaires lentes sont dues au développement dans le canal médullaire d’une lésion expansive. La localisation peut être intra ou extra médullaire. C’est une pathologie très fréquente dont le diagnostic est essentiellement clinique. L’imagerie par résonnance magnétique occupe une place incontournable dans le diagnostic de localisation et la recherche étiologique. En Europe l’étiologie tumorale est prépondérante. Dans ce travail nous apportons notre étude expérience à propos d’une série africaine de 97 patients.

## Méthodes

Il s’agit d’une étude rétrospective portant sur 97 observations colligées au service de radiologie du CHUN de Fann sur une période de 30 mois (du 08/03/10 au 29/09/12). Ont été inclus dans l’étude, tous les patients adressés pour un tableau de compression médullaire lente survenu dans un contexte non traumatique. L’âge moyen des patients était de 42,6 ans avec des extrêmes compris entre 04 mois et 85 ans. La sex-ratio était de 0,6. Les examens ont été réalisés sur un appareil d’IRM haut champ (1,5T Philips Achieva). Le protocole d’examen permettait la réalisation de séquence pondérées T1 sans avec injection de gado, T2, STIR et T2 DRIVE centrées sur les niveaux lésionnels ou les zones suspectes. Nous avons étudié la topographie des lésions (étage rachidien, compartiments canalaires) leur rehaussement et les critères d’orientation étiologiques.

## Résultats

### Résultats globaux

L’IRM avait trouvé des lésions de compression médullaire chez 61 patients soit 63% des cas. Chez les autres patients(36), l’IRM était normale dans 98% des cas, et a montré chez deux patients des tassements vertébraux ostéoporotiques.

### Etude topographique

#### Etages rachidiens (Figure 1, Figure 2, Figure 3)

##### Rehaussement des lésions

Les lésions d’allure infectieuse et tumorale se rehaussaient après injection de contraste excepté le kyste arachnoïdien.

**Figure 1:**
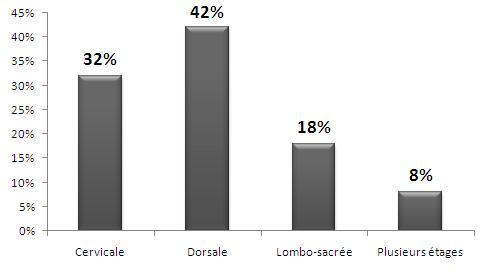
Répartition des lésions sur les étages rachidiens

**Figure 2:**
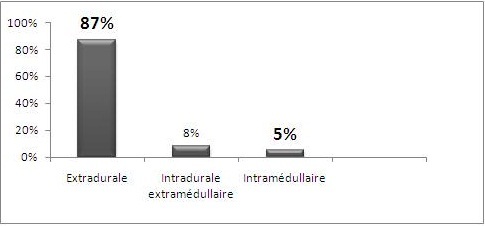
Distribution des lésions dans les compartiments canalaires

**Figure 3:**
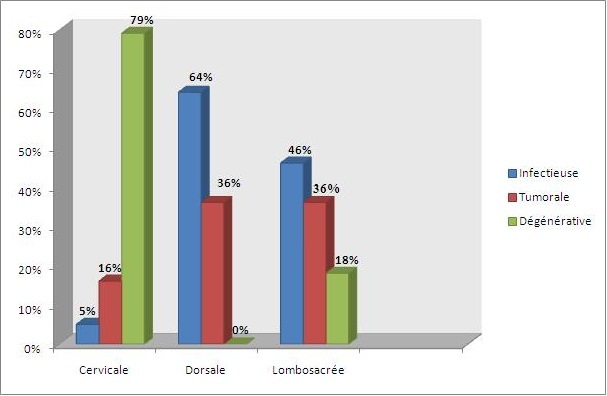
Répartition des différentes étiologies sur le rachis

### Orientations étiologiques

**Figure 4:**
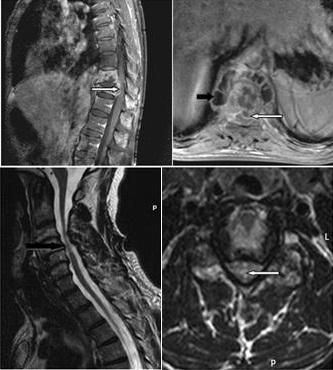
Compression médullaire. (A): coupes IRM sagittale et axiale pondérées en T1 avec injection de contraste, montrant une compression médullaire par épidurite antérieure réalisant un aspect en «embrase de rideau», (flèche blanche) et des collections abcédées péri-vertébrales (flèche noire); (B): coupes IRM sagittale et axiale pondérées en T2, montrant une cervico-uncarthrose (flèche noire) étagée responsable d’une myélopathie cervicarthrosique (flèche blanche) C4-C5 et C5-C6

**Figure 5:**
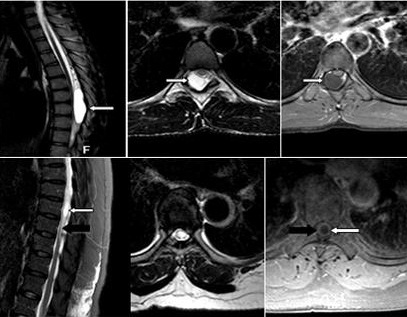
Compressions médullaires. (A): coupes IRM sagittale et axiales pondérées en T2 et T1, montrant une compression médullaire (flèche blanche) de D7 à D9, par un processus kystique intradural extra médullaire non rehaussé par le contraste (kyste arachnoïdien). (B): coupes IRM axiale et sagittale T2 STIR et axiale T1 avec injection de gadolinium montrant un processus expansif intramédulaire (flèche blanche) à hauteur de T8 bordé par un œdème médullaire(flèche noire) étendu de T5 à T10 (myélite infectieuse avec image d’abcédation)

L’analyse minutieuse des différents examens IRM confrontée aux données clinico-biologiques nous a permis d’avoir une orientation étiologique des compressions médullaires non traumatiques. Les causes extradurales d’origine infectieuse représentaient 38% de toutes les étiologies. Il s’agissait des épidurites en rapport avec des spondylodiscites (Figure 4A). Tandis que les épidurites d’origine tumorale étaient présentes dans 30% des cas. La pathologie dégénérative était rencontrée dans 32% des cas (Figure 4B). Les étiologies intradurales extramédullaires étaient tumorales et représentaient 8% des cas avec 03 cas de méningiome, 01 cas de schwannome et 01 cas de kyste arachnoïdien (Figure 5A). Les lésions intramédullaires étaient rencontrées dans 05% des cas, avec 02 cas de lésions d’allure tumorale (ependymome versus astrocytome) et 01 cas de myélite abcédée (Figure 5B).

## Discussion

Ce travail a permis de montrer la sensibilité de l’IRM vertèbro-médullaire dans le diagnostic topographique et étiologique des compressions médullaires lentes non traumatiques. L’analyse séméiologique minutieuse des lésions a l’IRM a permis de faire une répartition topographique des lésions en fonction de leur siège rachidien, leurs localisations intracanalaires et surtout d’avoir une orientation étiologique. Ainsi elle a montré une prédilection de la pathologie infectieuse au niveau de la charnière dorso-lombaire, la pathologie dégénérative à l’étage cervical, tandis que la pathologie tumorale siège aussi bien à l’étage lombo-sacré qu’au niveau des autres étages. *N Sans*[1] et *Kremer S* [2] dans leur étude sur l’infection du rachis-spondylodiscite, ont montré une prédominance de la localisation cervico-dorsale de la pathologie infectieuse, alors que dans l’étude de *N cherif idrissi El ganouni* et al [3], la région lombaire était la plus touchée. L’atteinte pluri-étagée est l’apanage de la pathologie tumorale comme l’atteste les données de la littérature [3–6]. Les causes de compression médullaire extradurale étaient les plus fréquentes suivies de la localisation intradurale extra médullaire et de celle intra médullaire. Dans la littérature [7–10], le compartiment extradural était le plus touché. Khalil [11] et Lecouvet F [12] dans leur étude ont trouvé plus de 75% des lésions dans le compartiment extradural. Toutes les entités nosologiques étaient rencontrées mais avec une prédominance des infections (38%). Il s’agissait toutes des épidurites dues à l’extension intracanalaire des spondylodiscites. L’aspect IRM de ces spondylodiscites était caractérisé par un hypersignal sur les séquences pondérées en T2 du disque intervertébral associé à un hypersignal en T2 des corps vertébraux adjacents. En séquence pondérée T1 il existe un hyposignal du disque et des plateaux adjacents. L’abcès para-vertébral présente un rehaussement de la coque après injection de gadolinium. Ces aspects sont retrouvés chez nos patients qui avaient un âge moyen de 42,6 ans, avec une prédominance masculine. *Ouboukhlik K* et al [13] dans leur série portant sur 100 cas de compressions médullaires lentes, ont trouvé un âge moyen de 36 ans avec une prédominance masculine. Dans une étude Tanzanienne [14], plus de la moitié des patients présentaient une compression médullaire d’origine infectieuse, mal de Pott en particulier. Ces données trouvent une explication du fait du bas niveau de vie socio-économique et culturelle et l’absence de couverture sanitaire.

La pathologie dégénérative représentait 32% des causes de compression médullaire extradurale dont une était compliquée de myélopathie cervicarthrosique. Ces lésions étaient rencontrées chez les sujets âgés. Les lésions tumorales, dans notre série, représentaient 30% de toutes les étiologies. Elles étaient dues à des épidurites métastatiques primitives. L’atteinte épidurale était responsable d’une amputation de l’espace graisseux épidural avec effet de masse sur les structures sous-arachnoïdiennes et médullaires. Les séquences T1 injectées avec suppression du signal graisseux délimitent souvent mieux l’infiltration épidurale. Dans notre série et l’étude marocaine [15], les épidurites infectieuses sont de loin supérieures aux épidurites métastatiques et elles ne touchent que la population adulte jeune contrairement aux séries européennes où les épidurites métastatiques représentent 90% des causes extradurales [16–20] et touchent une population plus âgée. Par contre en Europe, l’âge avancé et la prédominance des épidurites métastatiques s’expliquent par la prévalence élevée des tumeurs ostéophiles (poumon et prostate) qui sont une pathologie du sujet âgé. Les causes intradurales extramédullaires étaient toutes d’allure tumorale. Il s’agissait de méningiome, de schwannome et de kyste arachnoïdien. Les aspects IRM étaient caractéristiques. Ce sont les principales lésions rencontrées dans cet espace médullaire. Ces résultats corroborent avec les données de la littérature [5, 8]. Dans notre série aucun cas de métastase leptoméningée n’a été isolée. Ceci pourrait s’expliquer par le fait que ces lésions sont le plus souvent secondaires aux médulloblastomes, glioblastomes intracrâniens et rarement aux cancers viscéraux [5], causes non rencontrées dans notre étude. Les lésions intramédullaires étaient rares (05%). Un cas de myélite abcédée était isolé dans un contexte de spondylodiscite multifocale. Il s’agit d’une pathologie rare comme l’atteste les données de la littérature où son incidence est estimée à 01 cas par an [5, 18]. Il survient le plus souvent sur un terrain d’immunodépression. Les autres lésions rencontrées étaient d’allure tumorale et leurs aspects IRM n’étaient pas typiques. Un épendymome ou un astrocytome était évoqué. Ceci s’explique par le fait que le diagnostic différentiel de ces deux entités n’est pas aisé [5, 8, 19]. Un examen anatomopathologique nous aurait permis de poser le diagnostic positif de même que la spectro IRM. Ce qui constituait un des billais de notre étude. Les compressions médullaires lentes sont des lésions graves dont le pronostic fonctionnel dépend de la précocité du diagnostic et de l’étiologie. Les garants d’un meilleur pronostic reposent sur un traitement médical adéquat précoce de toute spondylodiscite et d’une laminectomie décompressive avant le stade myélomalacie.

## Conclusion

Les compressions médullaires lentes non traumatiques constituent un problème diagnostique et de prise en charge thérapeutique. La particularité du profil étiologique des compressions médullaires en Afrique sub-saharienne est la prédominance des épidurites infectieuses et la fréquence relative des épidurites métastatiques comparée aux séries occidentales. L’IRM vertébro-médullaire occupe une place capitale dans le diagnostic positif, topographique et étiologique des compressions médullaires.

### Etat des connaissances actuelle sur le sujet

- Le diagnostic des compressions médullaires lentes non traumatiques est essentiellement clinique;
- Les étiologies sont dominées en Europe par la pathologie tumorale.

### Contribution de notre étude à la connaissance

- En Afrique Sub-Saharienne les étiologies sont dominées par la pathologie infectieuse en particulier la tuberculose;
- La pathologie touche l’adulte jeune avec un âge moyen de 42 ans;
- L’IRM vertèbro-médullaire est l’examen de choix.
